# Supplementary figures and images for: Wavelet transform-based mode decomposition for EEG signals under general anesthesia
Source: PeerJ. 2024 Nov 15;12:e18518. doi: 10.7717/peerj.18518 (PMC11572389; doi:10.7717/peerj.18518)

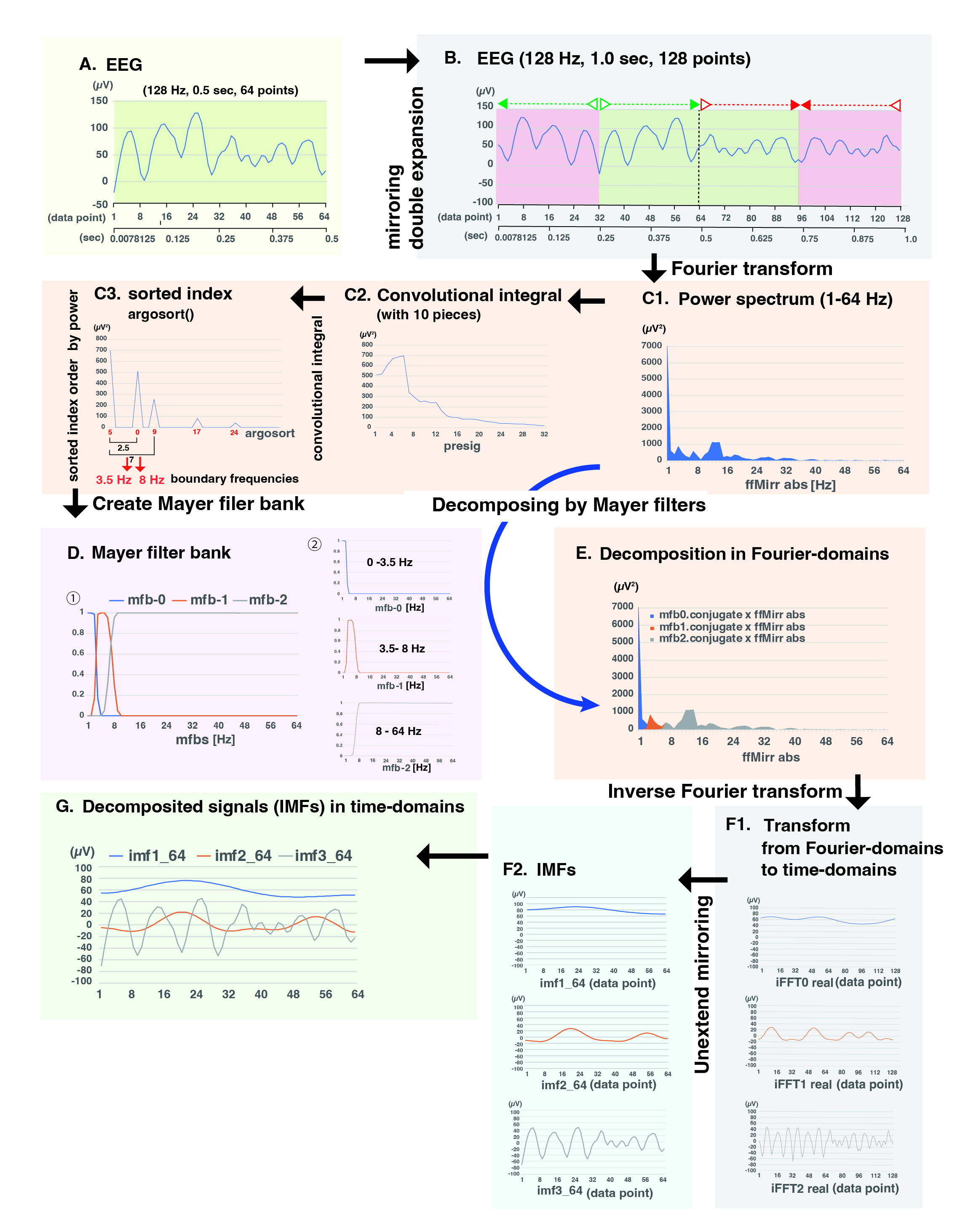

Supplement: Supplemental Information 1 — (A) An initial EEG wave (128 Hz, 0.5 s, 64 data points). (B) mirroring the double expansion of the original EEG wave (128 data points). (C) Fast Fourier transform. 1. Power spectrum, 2. Convolutional integral, and 3. Sorted index by argosoft() function. (D) Creation of Mayer filter bank (mfb-0, mfb-1, and mfb-2). (E) Decomposition in the frequency domain. (F) Inverse Fourier transform. 1. Inverse Fourier transform from the frequency domain to the time domain. 2. Un-mirroring to intrinsic mode functions (IMFs), (G). Decomposition into IMFs in time-domains. [file peerj-12-18518-s001.jpg]

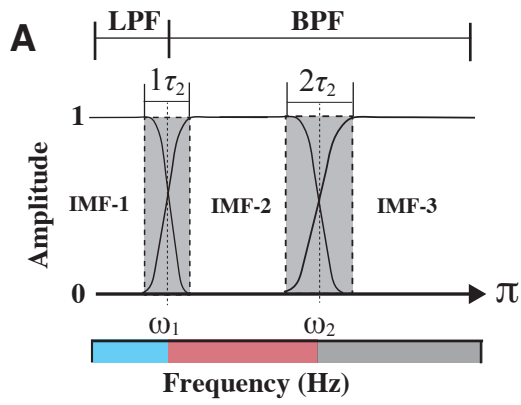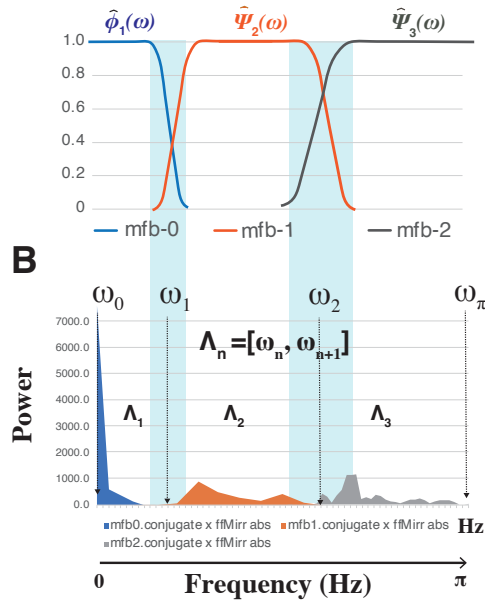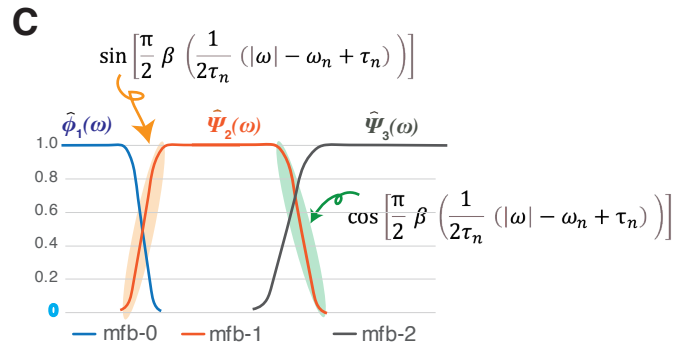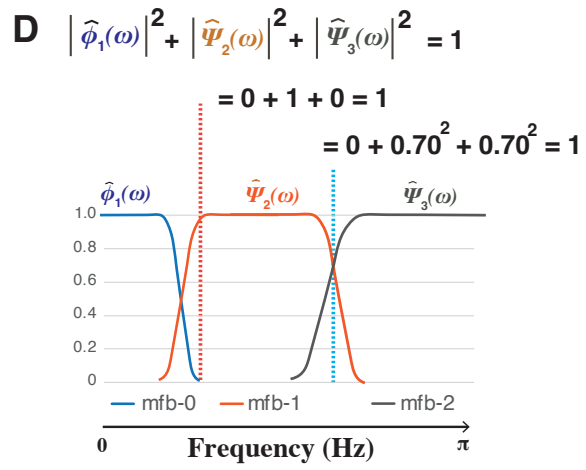

Supplement: Supplemental Information 2 — (A) ω = ωii=1,2,...,N (N denotes the number of maxima, and also, the number of filter banks.Assuming the frequency domain [0, π] is divided into N consecutive segments, we need to extract N-1 boundaries excluding 0 and π (This figure shows the case of N = 6). (B) To find the boundary in the EWT, local maxima in the spectrum are found and sorted in descending order, and the boundary is defined as the average between consecutive maxima. Let ω n be the limit between each segment (where ω0 = 0 and ωn = π), and denote each segment by Λn = [ωn−1, ωn], then ${\mathop{\cup }\nolimits }_{n-1}^{N}{\Lambda }_{n}=[0,\pi ]$∪n−1NΛn=0,π. (C) A scheme explaining Eqs. (2) and (3). (D) A tight frame constructed by Meyer’ s wavelet with a set of ϕ1(t), ψn(t)Nn=1 explaining (7). φ(): scale function, ψ (): wavelet function, β: beta function β (x) = x4(35 − 84x + 70x2 + 20x3) ((5)), τ : transition phase, ω : the limit between each segment (where ω0 = 0 and ωn = π). BPF, band-pass filter, IMF, intrinsic mode function; LPF, low-pass filter; Mfb, Meyer wavelet filter bank. [file peerj-12-18518-s002.pdf]

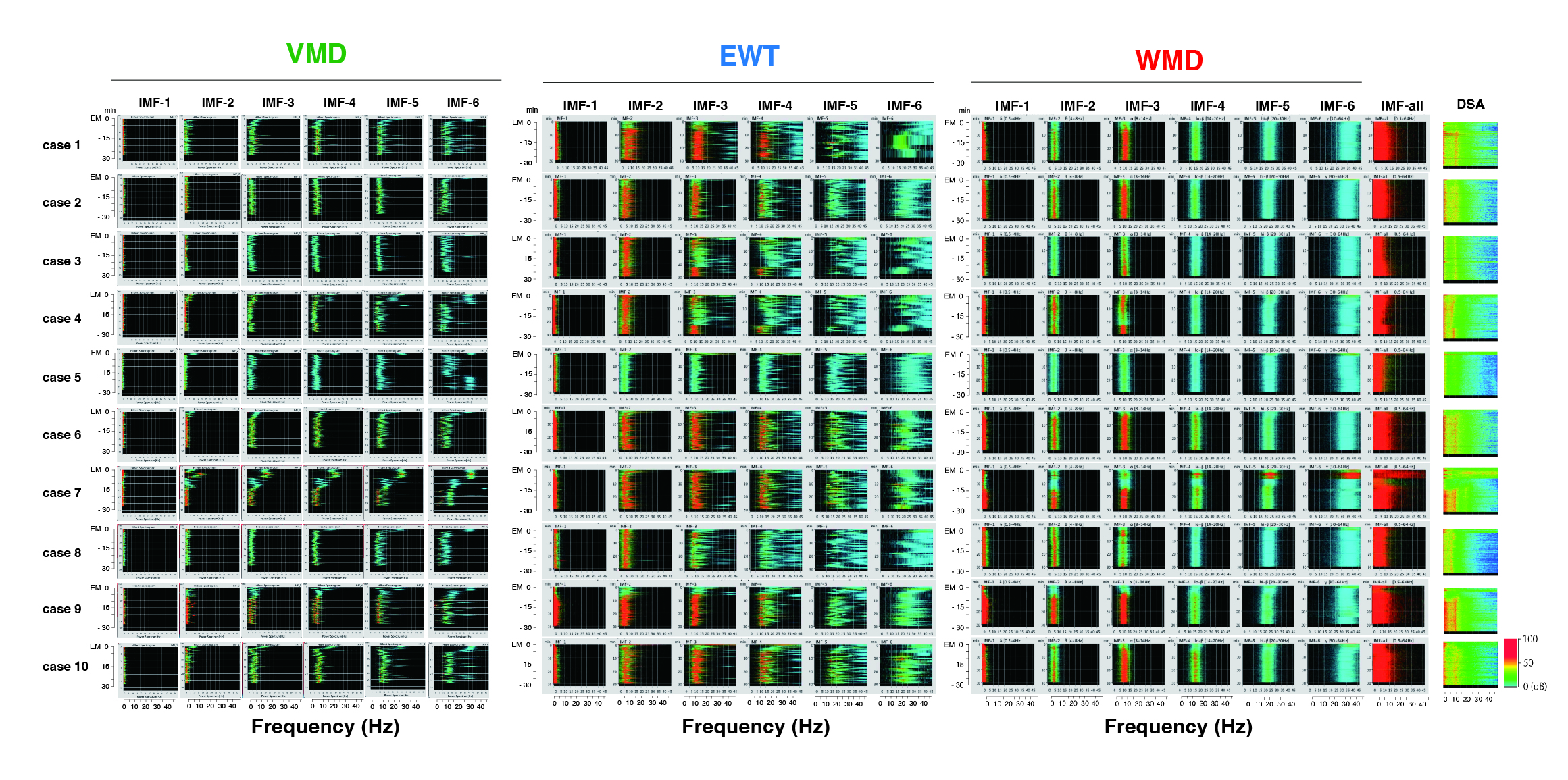

Supplement: Supplemental Information 3 — IMFs 1–6, a summed signal composed of all IMFs (IMF-all, which is the same as the initial EEG), and color DSAs for 30 min before emergence in all ten patients (as screen capture images from the EEG Mode Decompositor software) are shown. [file peerj-12-18518-s003.jpg]

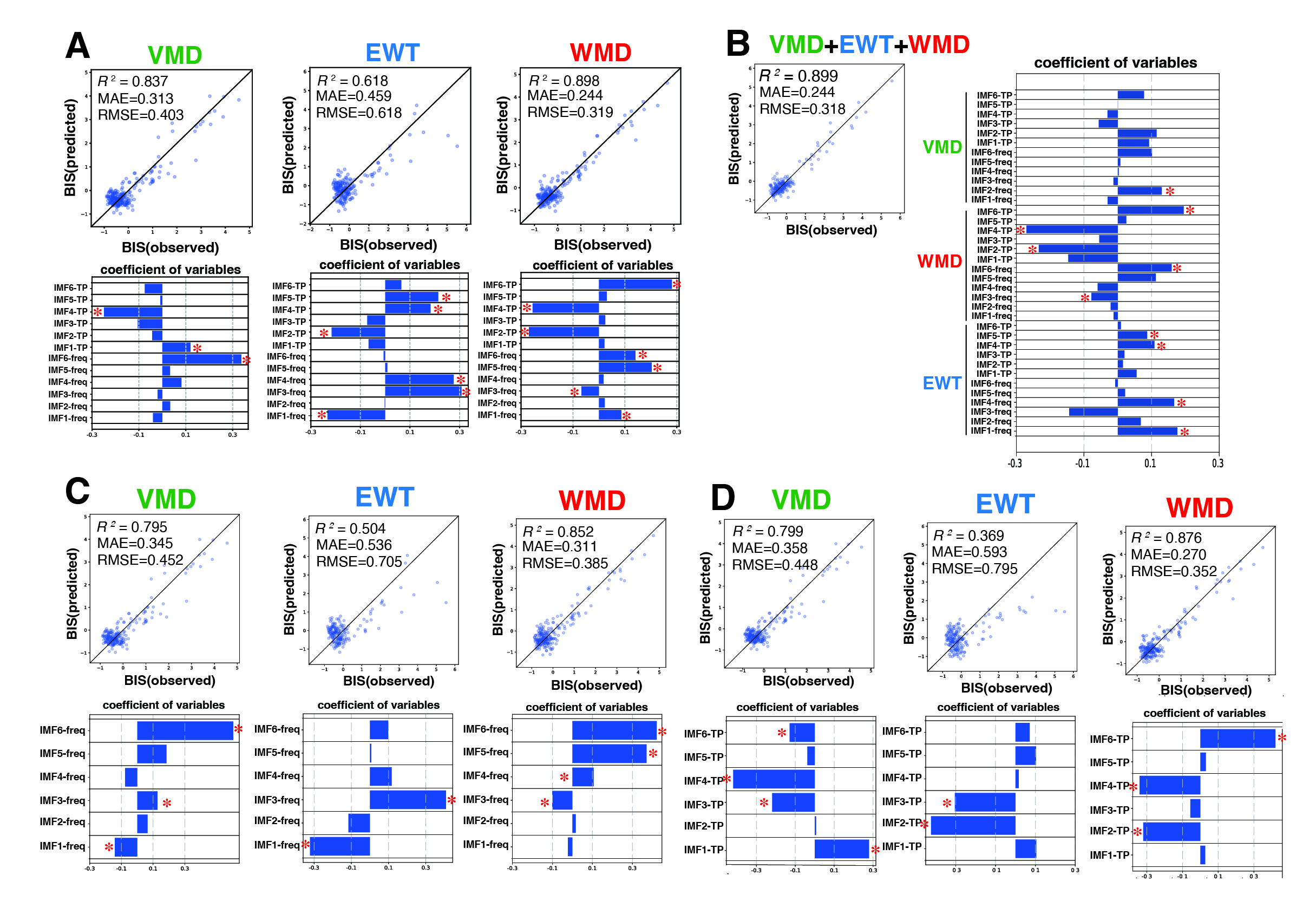

Supplement: Supplemental Information 4 — (A) In the in VMD, EWT, or WMD, using 6 median values of the central frequencies and 6 total powers (TPs) as explanatory variables. (B) Using all parameters of the IMFs derived from the VMD+EWT+WMD as explanatory variables. (C) In the three different mode decomposition using only 6 median values of the central frequencies as explanatory variables, and (D) in the three different mode decomposition using only 6 median values of total powers as explanatory variables. The EEG data were obtained from the last 30 min before emergence in ten patients who received sevoflurane general anesthesia. MAE, mean absolute error; RMSE, root mean squared error; freq, central frequency; TP, total power; ∗p < 0.05. [file peerj-12-18518-s004.jpg]
