# Supplementary material for: Wavelet transform-based mode decomposition for EEG signals under general anesthesia
Source: PeerJ. 2024 Nov 15;12:e18518. doi: 10.7717/peerj.18518 (PMC11572389; doi:10.7717/peerj.18518)
Supplement: Supplemental Information 7 [file peerj-12-18518-s007.pdf]

EWT_python_code

May 14, 2024

1 Empirical Wavelet Transform -1 : Python 3.8

2 Maintenance Phase

Feb 10, 2023 by Teiji Sawa, Department of Anesthesiology, Kyoto Prefectural University of Medicine

[ ]:
```
import numpy as np
import pandas as pd
import matplotlib.pyplot as plt
import matplotlib.patches as patches
from scipy.fftpack import fft
from scipy import signal as sig #Signal function import from SciPy package
from scipy import ndimage

from IPython.display import set_matplotlib_formats
set_matplotlib_formats('svg')
```

3 try EEG waveform

[ ]:
```
# df = pd.read_csv('eeg_1024.csv')
```

[ ]:
```
df_tsv = pd.read_table('./Yamada_Sev_DataSet/Sev_7_eeg_bis_20220629_EME30min.
 ↪tsv')
```

[ ]:
```
df_tsv.head(5)
```

[ ]:
```
df_tsv.tail(5)
```

[ ]:
```
cols = ['Time', 'eeg']
df_eeg = pd.DataFrame(data=None, index=[], columns=cols)
```

[ ]:
```
#for row in range(0, 4682):
    # Import only 1024 lines of data. Since 8 lines / second, 128 seconds = 2␣
 ↪minutes 8 seconds
    # Number of data = 16,384
    # By playing with the range here, you can change which brain waves you can␣
 ↪take out.
```

```
#    t_tmp = df_tsv.iat[row,1]
#    for column in range(2, 18):
#              eeg_tmp = df_tsv.iat[row, column]
#              data_tmp = pd.Series( [t_tmp, eeg_tmp], index=df_eeg.columns )
#              df_eeg = df_eeg.append(data_tmp, ignore_index=True)

cols = ['Time', 'eeg']
temp_data = []  # Initialization

for row in range(len(df_tsv)):
#for row in range(0, 2048):
#for row in range(0, 4682):
#for row in range(0, 4696):
    t_tmp = df_tsv.iat[row, 1]
    for column in range(2, 18):
        eeg_tmp = df_tsv.iat[row, column]
        data_tmp = [t_tmp, eeg_tmp]  # add data as list
        temp_data.append(data_tmp)

# temp_data Making DataFrame from list
df_eeg = pd.DataFrame(temp_data, columns=cols)
```

[ ]:
```
eeg_tmp
```

[ ]:
```
df_eeg
```

[ ]:
```
df_eeg.to_csv("./data_eeg_ewt2/eeg_linealized_eeg_bis_std2_1.csv")
```

[ ]:
```
# Place brain wave data in the data frame
time = df_eeg['Time'].values
eeg = df_eeg['eeg'].values
```

[ ]:
```
# Time-series graph display of EEG data
fig, ax = plt.subplots(figsize=(14,6))
plt.plot(eeg)
plt.savefig('data_eeg_ewt2/EWT_eeg_1.svg.svg')
```

[ ]:
```
eeg_ip = df_eeg['eeg'].values
```

[ ]:
```
#maintenance
eeg_512 = eeg_ip[0:512]
time_512 = time[0:512]
#eeg_512 = eeg_ip[5000:5512]
#time_512 = time[5000:5512]

#Yamochi paper=>
```

```
#eeg_512 = eeg_ip[3000:3512]
#time_512 = time[3000:3512]

#transition
#eeg_512 = eeg_ip[60000:60512]
#time_512 = time[60000:60512]

#emergence
#eeg_512 = eeg_ip[66719:67231]
#time_512 = time[66719:67231]

#eeg_512 = eeg_ip[64719:65231]
#time_512 = time[64719:65231]
```

[ ]:
```
# Time-series graph display of EEG data that complements and corrects missing␣
↪values
fig, ax = plt.subplots(figsize=(14,6))
plt.ylim(-100, 100)
plt.plot(eeg_512[0:512], linewidth =0.5)
plt.savefig('data_eeg_ewt2/EWT_eeg512_2.svg')
```

[ ]:
```
N=len(eeg_512[0:511]) # total data points
f_s = 128 # sampling frequency
T = 1.0/f_s  # data length
s_rate = 1/f_s # sampling rate
```

[ ]:
```
from scipy.fftpack import fft

xfft = np.linspace(0, int(1.0/(2.0*T)), int(N/2))
yfft=fft(eeg_512[0:511])
```

[ ]:
```
plt.plot(xfft, 2.0/N*abs(yfft[0:int(N/2)]), linewidth =0.5, ␣
↪label="periodogram")
plt.legend(bbox_to_anchor=(1, 1), loc='upper right', borderaxespad=1,␣
↪fontsize=12)
plt.grid()
plt.yscale("log")
plt.savefig('data_eeg_ewt2/eeg512_fft_2.svg')
```

4 Divide into 6 IMFs

[ ]:
```
#Emprical Wavelet Transform
def EWT1D(f, Fs, N):

    ff = np.fft.fft(f)
    ff = abs(ff[0:int(np.ceil(ff.size/2))])
```

```
#EWT_Boundaries_Detect()
boundaries = EWT_Boundaries_Detect(ff,N)
#print("boundaries=:", type(boundaries))
#print("boundaries=:", boundaries)
#boundaries = [8.0, 13.0]
#boundaries = [4.0, 8.0, 12.0, 20.0, 30.0]
#print("boundaries=:", type(boundaries))
print("boundaries[0]=:", boundaries[0])
print("boundaries[1]=:", boundaries[1])
print("boundaries[2]=:", boundaries[2])
print("boundaries[3]=:", boundaries[3])
print("boundaries[4]=:", boundaries[4])
#print("boundaries[5]=:", boundaries[5])

#print("boundaries=:", boundaries.size)
#for i in range(boundaries.size):
    #print("border[i]=:", boundaries[i])
print("Sampling Hz/2=:", round(Fs/2))

for i in range(len(boundaries)):
    #boundaries[i] = boundaries[i]*np.pi/round(ff.size)
    boundaries[i] = boundaries[i]*np.pi/round(Fs/2)

print("ff.size=:", round(ff.size))
print("boundaries_2[0]=:", boundaries[0])
print("boundaries_2[1]=:", boundaries[1])
print("boundaries_2[2]=:", boundaries[2])
print("boundaries_2[3]=:", boundaries[3])
print("boundaries_2[4]=:", boundaries[4])

#Filtering
#Mirroring
ltemp = int(np.ceil(f.size/2))
fMirr =  np.append(np.flip(f[0:ltemp-1],axis = 0),f)
fMirr = np.append(fMirr,np.flip(f[-ltemp-1:-1],axis = 0))

#FFT
ffMirr = np.fft.fft(fMirr)

#MEWT_Meyer_FilterBank()
mfb=EWT_Meyer_FilterBank(boundaries,ffMirr.size)

ewt = np.zeros(mfb.shape)
for k in range(mfb.shape[1]):
    ewt[:,k] = np.real(np.fft.ifft(np.conjugate(mfb[:,k])*ffMirr))
```

```
    ewt = ewt[ltemp-1:-ltemp,:]

    return ewt, mfb, boundaries
```

[ ]:
```
#EWT_Boundaries_Detect()

def EWT_Boundaries_Detect(ff,N):

    #Normalization
    regFilter = np.ones(10)/10
    presig = np.convolve(ff,regFilter,mode = 'same') #convolution integral

    #boundaries
    boundaries = LocalMax(presig,N)

    return boundaries+1
```

[ ]:
```
#LocalMax()

def LocalMax(ff, N):

    N=N-1
    locmax = np.zeros(ff.size)
    for i in np.arange(1,ff.size-1):
        if ff[i-1]<ff[i] and ff[i]>ff[i+1]:
            locmax[i] = ff[i]

    N = min(N,locmax.size)

    maxidxs = np.sort(locmax.argsort()[::-1][:N])

    #middle point between consecutive maxima
    bound = np.zeros(N)
    for i in range(N):
        if i == 0:
            a = 0
        else:
            a = maxidxs[i-1]
        bound[i] = (a + maxidxs[i])/2

    return bound
```

[ ]:
```
#Meyer Filter Bank

def EWT_Meyer_FilterBank(boundaries,Nsig):

    Npic = len(boundaries)
```

```python
    gamma = 1
    for k in range(Npic-1):
        r = (boundaries[k+1]-boundaries[k])/ (boundaries[k+1]+boundaries[k])
        if r < gamma:
            gamma = r
    r = (np.pi - boundaries[Npic-1])/(np.pi + boundaries[Npic-1])
    if r <gamma:
        gamma = r
    gamma = (1-1/Nsig)*gamma

    mfb = np.zeros([Nsig, Npic+1])

    # the empirical scaling function)
    Mi=int(np.floor(Nsig/2))
    w_pre = np.linspace(0,2*np.pi - 2*np.pi/Nsig,num = Nsig)
    w=np.fft.fftshift(w_pre)
    w[0:Mi]=-2*np.pi+w[0:Mi]
    aw=abs(w)

    yms=np.zeros(Nsig)
    an=1./(2*gamma*boundaries[0])
    pbn=(1.+gamma)*boundaries[0]
    mbn=(1.-gamma)*boundaries[0]

    for k in range(Nsig):
       if aw[k]<=mbn:
           yms[k]=1
       elif ((aw[k]>=mbn) and (aw[k]<=pbn)):
           yms[k]=np.cos(np.pi*EWT_beta(an*(aw[k]-mbn))/2)

    yms=np.fft.ifftshift(yms)

    mfb[:,0] = yms

    for k in range(Npic-1):
        mfb[:,k+1] = EWT_Meyer_Wavelet(boundaries[k],boundaries[k+1], gamma,␣
 ↪Nsig)

    mfb[:,Npic] = EWT_Meyer_Wavelet(boundaries[Npic-1],np.pi, gamma, Nsig)

    return mfb
```

[ ]:
```python
#Beta function()

def EWT_beta(x):
```

```
    if x<0:
        bm=0
    elif x>1:
        bm=1
    else:
        bm=(x**4)*(35.-84.*x+70.*(x**2)-20.*(x**3))
    return bm
```

[ ]:
```
#EWT_Meyer_Wavelet()

def EWT_Meyer_Wavelet(wn, wm, gamma, Nsig):

    Mi=int(np.floor(Nsig/2))
    w=np.fft.fftshift(np.linspace(0,2*np.pi - 2*np.pi/Nsig,num = Nsig))

    w[0:Mi]=-2*np.pi+w[0:Mi]
    aw=abs(w)

    ymw=np.zeros(Nsig)
    an=1./(2*gamma*wn)
    am=1./(2*gamma*wm)
    pbn=(1.+gamma)*wn
    mbn=(1.-gamma)*wn
    pbm=(1.+gamma)*wm
    mbm=(1.-gamma)*wm

    for k in range(Nsig):
       if ((aw[k]>=pbn) and (aw[k]<=mbm)):
           ymw[k]=1
       elif ((aw[k]>=mbm) and (aw[k]<=pbm)):
           ymw[k]=np.cos(np.pi*EWT_beta(am*(aw[k]-mbm))/2)
           #print("cos=:",ymw[k])
       elif ((aw[k]>=mbn) and (aw[k]<=pbn)):
           ymw[k]=np.sin(np.pi*EWT_beta(an*(aw[k]-mbn))/2)
           #print("sin=:", ymw[k])
    ymw=np.fft.ifftshift(ymw)
    return ymw
```

[ ]:
```
sig = eeg_512[0:512]
ewt2, mfb2, boundaries2 = EWT1D(sig, Fs = 128, N = 6)
```

[ ]:
```
plt.plot(ewt2)
plt.savefig('data_eeg_ewt2/EWT_imf_1.svg')
```

[ ]:
```
#Get EEG, 512 data point (128Hz, 4 sec sanpling freq = 128Hz
#T2 = 64
T2 = 512
```

```
Fs =128
t2 = np.arange(1,T2+1)/Fs
Ts = 1.0/Fs
```

[ ]:
```
xfft_1 = np.linspace(0, int(1.0/(2.0*Ts)), int(T2/2))
yfft_1 =fft(ewt2[:,0])
```

[ ]:
```
plt.plot(xfft_1, 2.0/T2*abs(yfft_1[0:int(T2/2)]), linewidth =0.5, ␣
  ↪label="periodogram")
plt.legend(bbox_to_anchor=(1, 1), loc='upper right', borderaxespad=1,␣
  ↪fontsize=12)
plt.grid()
plt.savefig('data_eeg_ewt2/EWT_yfft1_1.svg')
#plt.yscale("log")
```

[ ]:
```
xfft_2 = np.linspace(0, int(1.0/(2.0*Ts)), int(T2/2))
yfft_2 =fft(ewt2[:,1])
```

[ ]:
```
plt.plot(xfft_2, 2.0/T2*abs(yfft_2[0:int(T2/2)]), linewidth =0.5, ␣
  ↪label="periodogram")
plt.legend(bbox_to_anchor=(1, 1), loc='upper right', borderaxespad=1,␣
  ↪fontsize=12)
plt.grid()
plt.savefig('data_eeg_ewt2/EWT_yfft2_1.svg')
#plt.yscale("log")
```

[ ]:
```
xfft_3 = np.linspace(0, int(1.0/(2.0*Ts)), int(T2/2))
yfft_3 =fft(ewt2[:,2])
```

[ ]:
```
plt.plot(xfft_3, 2.0/T2*abs(yfft_3[0:int(T2/2)]), linewidth =0.5, ␣
  ↪label="periodogram")
plt.legend(bbox_to_anchor=(1, 1), loc='upper right', borderaxespad=1,␣
  ↪fontsize=12)
plt.grid()
plt.savefig('data_eeg_ewt2/EWT_yfft3_1.svg')
#plt.yscale("log")
```

[ ]:
```
xfft_4 = np.linspace(0, int(1.0/(2.0*Ts)), int(T2/2))
yfft_4 =fft(ewt2[:,3])
```

[ ]:
```
plt.plot(xfft_4, 2.0/T2*abs(yfft_4[0:int(T2/2)]), linewidth =0.5, ␣
  ↪label="periodogram")
plt.legend(bbox_to_anchor=(1, 1), loc='upper right', borderaxespad=1,␣
  ↪fontsize=12)
plt.grid()
plt.savefig('data_eeg_ewt2/EWT_yfft4_1.svg')
#plt.yscale("log")
```

[ ]:
```
xfft_5 = np.linspace(0, int(1.0/(2.0*Ts)), int(T2/2))
yfft_5 =fft(ewt2[:,4])
```

[ ]:
```
plt.plot(xfft_5, 2.0/T2*abs(yfft_5[0:int(T2/2)]), linewidth =0.5, 
 ↪label="periodogram")
plt.legend(bbox_to_anchor=(1, 1), loc='upper right', borderaxespad=1,
 ↪fontsize=12)
plt.grid()
plt.savefig('data_eeg_ewt2/EWT_yfft5_1.svg')
#plt.yscale("log")
```

[ ]:
```
xfft_6 = np.linspace(0, int(1.0/(2.0*Ts)), int(T2/2))
yfft_6 =fft(ewt2[:,5])
```

[ ]:
```
plt.plot(xfft_6, 2.0/T2*abs(yfft_6[0:int(T2/2)]), linewidth =0.5, 
 ↪label="periodogram")
plt.legend(bbox_to_anchor=(1, 1), loc='upper right', borderaxespad=1,
 ↪fontsize=12)
plt.grid()
plt.savefig('data_eeg_ewt2/EWT_yfft6_1.svg')
#plt.yscale("log")
```

[ ]:
```
#pip install PyWavelets

import pywt
from scipy.fftpack import fft
```

[ ]:
```
wavlist = pywt.wavelist(kind='all')
print(wavlist)
```

[ ]:
```
wavelet_type = 'dmey'
wav = pywt.Wavelet(wavelet_type)

int_psi, x = pywt.integrate_wavelet(wav, precision=2)
print(len(int_psi))
plt.plot(x, int_psi)
plt.savefig('data_eeg_ewt2/EWT_wavelet_20220827_dmey1.svg')
plt.show()
```

[ ]:
```
f_s = 128
T = 1.0/f_s
N2=len(int_psi)
xfft = np.linspace(0, int(1.0/(2.0*T)), int(N2/2))
yfft=fft(int_psi)
```

[ ]:
```

```

```
plt.plot(xfft, 2.0/N2*abs(yfft[0:int(N2/2)]), linewidth =0.5,  label="Meyer")
plt.legend(bbox_to_anchor=(1, 1), loc='upper right', borderaxespad=1,␣
 ↪fontsize=12)
plt.grid()
plt.savefig('data_eeg_ewt2/EWT_Meyer_wavelet_fft1_1.svg')
```

[ ]:
```
plt.plot(xfft, 2.0/N2*abs(yfft[0:int(N2/2)]), linewidth =0.5,  label="Meyer")
plt.legend(bbox_to_anchor=(1, 1), loc='upper right', borderaxespad=1,␣
 ↪fontsize=12)
plt.grid()
plt.xlim(0,50)
plt.savefig('data_eeg_ewt2/EWT_Meyer_wavelet_fft2_1.svg')
```

[ ]:
```
plt.plot(xfft, 2.0/N2*abs(yfft[0:int(N2/2)]), linewidth =0.5,  label="Meyer")
plt.legend(bbox_to_anchor=(1, 1), loc='upper right', borderaxespad=1,␣
 ↪fontsize=12)
plt.grid()
plt.xlim(0,50)
plt.yscale("log")
plt.savefig('data_eeg_ewt2/EWT_Meyer_wavelet_fft3_1.svg')
```

[ ]:
```
int_psi, x = pywt.integrate_wavelet(wav, precision=4)
print(len(int_psi))
plt.plot(x, int_psi)
plt.savefig('data_eeg_ewt2/EWT_wavelet_20220827_dmey2.svg')
plt.show()
```

[ ]:
```
f_s = 128
T = 1.0/f_s
N2=len(int_psi)
xfft = np.linspace(0, int(1.0/(2.0*T)), int(N2/2))
yfft=fft(int_psi)
```

[ ]:
```
plt.plot(xfft, 2.0/N2*abs(yfft[0:int(N2/2)]), linewidth =0.5,  label="Meyer")
plt.legend(bbox_to_anchor=(1, 1), loc='upper right', borderaxespad=1,␣
 ↪fontsize=12)
plt.grid()
plt.savefig('data_eeg_ewt2/EWT_Meyer_wavelet_fft4_1.svg')
```

[ ]:
```
plt.plot(xfft, 2.0/N2*abs(yfft[0:int(N2/2)]), linewidth =0.5,  label="Meyer")
plt.legend(bbox_to_anchor=(1, 1), loc='upper right', borderaxespad=1,␣
 ↪fontsize=12)
plt.grid()
plt.xlim(0,15)
plt.savefig('data_eeg_ewt2/EWT_Meyer_wavelet_fft5_1.svg')
```

```
[ ]: plt.plot(xfft, 2.0/N2*abs(yfft[0:int(N2/2)]), linewidth =0.5,  label="Meyer")
     plt.legend(bbox_to_anchor=(1, 1), loc='upper right', borderaxespad=1,␣
      ↪fontsize=12)
     plt.grid()
     plt.xlim(0,15)
     plt.yscale("log")
     plt.savefig('data_eeg_ewt2/EWT_Meyer_wavelet_fft6_1.svg')
```

```
[ ]: import emd
```

```
[ ]: # Get the default configuration for a sift
     config = emd.sift.get_config('sift')
     # Adjust the threshold for accepting an IMF
     config['imf_opts/sd_thresh'] = 0.05
     imf = emd.sift.sift(sig)
```

```
[ ]: emd.plotting.plot_imfs(imf, cmap=True)
     plt.savefig('data_eeg_ewt2/EWT_emd_1.svg')
```

```
[ ]: emd.plotting.plot_imfs(ewt2, cmap=True)
     plt.savefig('data_eeg_ewt2/EWT_ewt2d_1.svg')
```

```
[ ]: sample_rate = 128
     seconds = 4
     num_samples = sample_rate*seconds
     time_vect = np.linspace(0, seconds, num_samples)
```

```
[ ]: IP, IF, IA = emd.spectra.frequency_transform(ewt2, sample_rate, 'hilbert')
```

```
[ ]: freq_edges, freq_bins = emd.spectra.define_hist_bins(0.1, 64, 128)
      #hht = emd.spectra.hilberthuang(IF, IA, freq_edges)

     freq_range = (0, 64, 128)  # 0 to 64Hz in 128 steps
     f, hht = emd.spectra.hilberthuang(IF, IA, freq_range, sum_time=False)
```

```
[ ]: freq_edges, freq_centres = emd.spectra.define_hist_bins(0, 100, 128, 'linear')

     # Amplitude weighted HHT per IMF
     f, spec_weighted = emd.spectra.hilberthuang(IF, IA, freq_edges, sum_imfs=False)

     # Unweighted HHT per IMF - we replace the instantaneous amplitude values with␣
      ↪ones
     f, spec_unweighted = emd.spectra.hilberthuang(IF, np.ones_like(IA), freq_edges,␣
      ↪sum_imfs=False)
```

```
[ ]: plt.figure(figsize=(10, 4))
     plt.subplots_adjust(hspace=0.4)
```

```
plt.subplot(121)
plt.plot(freq_centres, spec_unweighted)
plt.xticks(np.arange(10)*10)
plt.ylim(0, 450)
plt.xlim(0, 64)
plt.xlabel('Frequency (Hz)')
plt.ylabel('Count')
plt.title('unweighted\nHilbert-Huang Transform')

plt.subplot(122)
plt.plot(freq_centres, spec_weighted)
plt.xticks(np.arange(10)*10)
plt.xlim(0, 64)
plt.xlabel('Frequency (Hz)')
plt.ylabel('Power')
plt.title('IA-weighted\nHilbert-Huang Transform')
plt.legend(['IMF-1', 'IMF-2', 'IMF-3', 'IMF-4', 'IMF-5', 'IMF-6'],␣
 ↪frameon=False)
plt.savefig('data_eeg_ewt2/EWT_HHT_freq_1.svg')
```

[ ]:
```
fig = plt.figure(figsize=(6, 4))
emd.plotting.plot_hilberthuang(hht, time_vect, freq_bins,
                               time_lims=(0, 4), freq_lims=(0.1, 64), vmax=100,
                               fig=fig, log_y=False)
plt.savefig('data_eeg_ewt2/EWT_HHT_spectrum-1_12.svg')
```

[ ]:
```
fig = plt.figure(figsize=(6, 4))
emd.plotting.plot_hilberthuang(hht, time_vect, freq_bins,cmap='ocean_r',
                               time_lims=(0, 4), freq_lims=(0.1, 64), vmax=100,
                               fig=fig, log_y=False)
plt.savefig('data_eeg_ewt2/eme_EWT_HHT_spectrum-2_12.svg')
```

[ ]:
```
print(emd.plotting.plot_hilberthuang.__doc__)
```

[ ]:
```
fig = plt.figure(figsize=(6, 4))
emd.plotting.plot_hilberthuang(hht, time_vect, freq_bins, cmap='jet',
                               time_lims=(0, 4), freq_lims=(0.1, 64),vmax=1000,
                               fig=fig, log_y=False)
plt.savefig('data_eeg_ewt2/EWT_HHT_spectrum-3_12.svg')
```

[ ]:
```
fig = plt.figure(figsize=(6, 4))
emd.plotting.plot_hilberthuang(hht, time_vect, freq_bins, cmap='gist_heat_r',
                               time_lims=(0, 4), freq_lims=(0.1, 64),
                               fig=fig, log_y=False)
plt.savefig('data_eeg_ewt2/EWT_HHT_spectrum-4_12.svg')
```
