# Supplementary material for: Wavelet transform-based mode decomposition for EEG signals under general anesthesia
Source: PeerJ. 2024 Nov 15;12:e18518. doi: 10.7717/peerj.18518 (PMC11572389; doi:10.7717/peerj.18518)
Supplement: Supplemental Information 8 [file peerj-12-18518-s008.pdf]

```
import com.github.psambit9791.jdsp.signal.Convolution;

import java.util.Arrays;
import java.util.Comparator;
import java.util.Random;



//=========================================================
// Empirical Wavelet Transformation. EWT_ver01
// Processing Supported with JAVA
//    Aug 21, 2022
//    Teiji Sawa, Anesthesiology, Kyto Prefectural Univ. of Medicine
//    EWT Porting code from Python to Processing
//=========================================================
//Empirical Wavelet Transform implementation for 1D signals
//
//@author: Vinícius Rezende Carvalho
//Programa de pós graduação em engenharia elétrica - PPGEE UFMG
//Universidade Federal de Minas Gerais - Belo Horizonte, Brazil
//Núcleo de Neurociências - NNC
//=========================================================

class Ewt {

  //    signal    - the time domain signal (1D) to be decomposed
  //    K             - the number of modes to be recovered

  double signal2[];
  int K;
  int Fs = 128;

  // Constructor
  Ewt (double [] _signal, int _K) {
    signal2 = _signal;
    K = _K;
  }

  double [][] ewt(double [] _signal) {
    signal2 = _signal;
    //Emd emd = new Emd();
    //EmdDataImpl emdData = new EmdDataImpl();

    int data_N = int(signal2.length);
    ewt = EWT1D(signal2, Fs, K);

    //emd.emdCreate(emdData, data.length-15, order, 20, 0);
    //emd.emdDecompose(emdData, data);
    //imfs = emd.imfs(emdData);
    for (int k=0; k<K; k++) {
      for (int i=0; i<data_N; i++) {
        imfs[k][i] = ewt[i][k];
      }
    }
```

```
        return imfs;
    }
}

class Ewt2 {

    //    signal    - the time domain signal (1D) to be decomposed
    //    K             - the number of modes to be recovered

    double signal2[];
    int K;
    int Fs = 128;

    // Constructor
    Ewt2 (double [] _signal, int _K) {
        signal2 = _signal;
        K = _K;
    }

    double [][] ewt(double [] _signal) {
        signal2 = _signal;
        //Emd emd = new Emd();
        //EmdDataImpl emdData = new EmdDataImpl();

        int data_N = int(signal2.length);
        ewt = EWT1D2(signal2, Fs, K);

        //emd.emdCreate(emdData, data.length-15, order, 20, 0);
        //emd.emdDecompose(emdData, data);
        //imfs = emd.imfs(emdData);
        for (int k=0; k<K; k++) {
            for (int i=0; i<data_N; i++) {
                imfs[k][i] = ewt[i][k];
            }
        }
        return imfs;
    }
}

double[][] EWT1D(double f[], int Fs, int N) {
    //
=============================================================
    //         ewt,    mfb ,boundaries = EWT1D(f, N = 5):
    //           log  =  0,detect  =  "locmax",  completion  =  0,  reg  =  'average',  lengthFilter  =
10,sigmaFilter = 5
    //
=============================================================
    //         Perform the Empirical Wavelet Transform of f over N scales.
    //
    //         Inputs:
    //             -f: the 1D input signal
    //         Optional Inputs:
    //           -log: 0 or 1 to indicate if we want to work with ==> log = 0
    //                                   the log spectrum
```

```
//          -method: 'locmax','locmaxmin','locmaxminf'
//          -reg: 'none','gaussian','average'
//         -lengthFilter: width of the above filters (Gaussian or average)
//          -sigmaFilter: standard deviation of the above Gaussian filter
//          -N: maximum number of supports (modes or signal components)
//          -completion: 0 or 1 to indicate if we try to complete
//                                   or not the number of modes if the detection
//                                   find a lower number of mode than N
//        Outputs:
//          -ewt: contains first the low frequency component and
//                  then the successives frequency subbands
//          -mfb: contains the filter bank (in the Fourier domain)
//          -boundaries: vector containing the set of boundaries corresponding
//                            to the Fourier line segmentation (normalized between
//                            0 and Pi)
//        Original MATLAB Version:
//        Author: Jerome Gilles
//        Institution: UCLA - Department of Mathematics
//        Year: 2013
//        Version: 2.0
//
//        Python Version: Vinícius Rezende Carvalho - vrcarva@ufmg.br
//        Universidade Federal de Minas Gerais - Brasil
//        Núcleo de Neurociências
//
==========================================================

double f2[];
Complex f3_pre[];
Complex f3[];
double fMirr[];
double fMirr2[];
Complex[] ff1;
double[] ff2;
Complex[] ffMirr1;
Complex[] ffMirr2;
double[] boundaries;
int ltemp;
double[][] mfb;
double[][] ewt;

FastFourierTransformer fft4 = new
FastFourierTransformer(DftNormalization.STANDARD);
ff1 = fft4.transform(f, TransformType.FORWARD);

ff2 = new double[int(ceil(ff1.length/2))];
for (int i=0; i< int(ceil(ff1.length/2)); i++) {
  ff2[i] = ff1[i].abs();
}

boundaries = EWT_Boundaries_Detect(ff2, N);
for (int i=0; i<boundaries.length; i++) {
  //boundaries[i] = boundaries[i] * PI/round(ff2.length);
  //boundaries[i] = boundaries[i] * PI/32;
```

```
        boundaries[i] = (boundaries[i]-128/1028) * PI/(Fs/2);
    }

    //Filtering: extend the signal by mirroring to deal with boundaries
    ltemp = int(ceil(f.length/2));

    fMirr = new double[ltemp];
    for (int i=0; i<ltemp; i++) {
        //fMirr[i] = (float) f[ltemp-1-i];
        fMirr[i] = f[ltemp-1-i];
    }

    //for (int i=0; i<f.length ; i++) {
    //fMirr = append(fMirr, (float) f[i]);
    fMirr = append_double(fMirr, f);
    //}

    f2 = new double[ltemp];
    for (int i=0; i<ltemp; i++) {
        f2[i] = f[f.length-2-i];
    }

    //for (int i=0; i<f2.length ; i++) {
    fMirr = append_double(fMirr, f2);
    //}

    fMirr2 = new double[fMirr.length];
    for (int i=0; i<fMirr2.length; i++) {
        fMirr2[i] = fMirr[i];
    }

    FastFourierTransformer fft2 = new
FastFourierTransformer(DftNormalization.STANDARD);
    ffMirr1 = fft2.transform(fMirr2, TransformType.FORWARD);

    //build the corresponding filter bank
    mfb = EWT_Meyer_FilterBank(boundaries, ffMirr1.length);

    //filter the signal to extract each subband
    ewt = new double [mfb.length][mfb[0].length];
    for (int i=0; i<mfb.length; i++) {
        for (int j=0; j<mfb[0].length; j++) {
            ewt[i][j]= 0;
        }
    }

    f3_pre = new Complex[mfb.length];
    f3 = new Complex[mfb.length];

    for (int k=0; k<mfb[0].length; k++) {
        for (int i=0; i < mfb.length; i++) {
            f3_pre[i] = Complex.valueOf(mfb[i][k]);
            f3[i] = f3_pre[i].conjugate().multiply(ffMirr1[i]);
        }
```

```
    FastFourierTransformer fft3 = new
FastFourierTransformer(DftNormalization.STANDARD);
    ffMirr2 = fft3.transform(f3, TransformType.INVERSE);
    for (int i=0; i <mfb.length; i++) {
      ewt[i][k] = ffMirr2[i].getReal();
    }
  }

  for (int k=0; k<ewt[0].length; k++) {
    for (int i=0; i<(mfb.length-ltemp); i++) {
      ewt[i][k] = ewt[ltemp-1+i][k];
      //ewt = ewt[ltemp-1:-ltemp,:]
    }
  }
  //return(ewt, mfb, boundaries);
  return(ewt);
}

double[] EWT_Boundaries_Detect(double[] ff, int N) {
  //
==========================================================
================
  // def EWT_Boundaries_Detect(ff,log,detect, N, reg, lengthFilter,sigmaFilter):
  // log = 0,detect = "locmax", reg = 'average', lengthFilter = 10,sigmaFilter = 5
  //
==========================================================
================
  // """This function segments f into a certain amount of supports by using different
technics:
  //      - middle point between consecutive local maxima (default),
  //      - lowest minima between consecutive local maxima (locmaxmin),
  //      - lowest minima between consecutive local maxima of original spectrum
(locmaxminf),
  //
  //      Regularized version of the spectrum can be obtained by the
  //      following methods:
  //      - Gaussian filtering (its parameters are filter of width
  //        lengthFilter and standard deviation sigmaFilter)scalesp
  //      - Average filtering (its parameters are filter of width
  //        lengthFilter)
  //
  //      Note: the detected boundaries are given in term of indices
  //
  //      Inputs:
  //        -f: the function to segment
  //      Optional parameters:
  //        -log: 0 or 1 to indicate if we want to work with
  //                    the log of the ff
  //        -reg: 'none','gaussian','average'
  //        -lengthFilter: width of the above filters (Gaussian or average)
  //        -sigmaFilter: standard deviation of the above Gaussian filter
  //        -N: maximum number of supports (modes or signal components)
  //        -completion: 0 or 1 to indicate if we try to complete
```

```
    //                                                  or not the number of modes if the detection
    //                                                  find a lower number of mode than N
    //
    //          Outputs:
    //              -boundaries: list of detected boundaries
    //
    //
    //          Original MATLAB version:
    //          Author: Jerome Gilles + Kathryn Heal
    //          Institution: UCLA - Department of Mathematics
    //          Year: 2013
    //          Version: 2.0
    //
    //          Python Version: Vinícius Rezende Carvalho - vrcarva@ufmg.br
    //          Universidade Federal de Minas Gerais - Brasil
    //          Núcleo de Neurociências
    //          """

    double [] regFilter;
    double [] boundaries;
    double [] presig;

    regFilter = new double [10];
    //Regularization
    for (int i=0; i<10; i++) {
        regFilter[i] = 1.0/10;
    }

    String mode = "same"; //Can be "valid", "same"
    Convolution con = new Convolution(ff, regFilter);
    presig = con.convolve(mode);

    //Boundaries detection: Mid-point between two consecutive local maxima computed on
the regularized spectrum
    boundaries = LocalMax(presig, N);
    for (int i=0; i < boundaries.length; i++) {
        boundaries[i] = boundaries[i] + 0.125;
    }
    return(boundaries);
}

double [] LocalMax(double[] ff, int N) {

//==========================================================
=========
    // def LocalMax(ff, N):

//==========================================================
=========
    //    bound = LocalMax(f,N)
    //
    //          This function segments f into a maximum of N supports by taking
    //          the middle point between the N largest local maxima.
    //          Note: the detected boundaries are given in term of indices
```

```
    //
    //      Inputs:
    //          -f: the function to segment
    //          -N: maximal number of bands
    //
    //      Outputs:
    //          -bound: list of detected boundaries
    //
    //      Original MATLAB version:
    //      Author: Jerome Gilles + Kathryn Heal
    //      Institution: UCLA - Department of Mathematics
    //      Year: 2013
    //      Version: 1.0
    //
    //      Python Version: Vinícius Rezende Carvalho - vrcarva@ufmg.br
    //      Universidade Federal de Minas Gerais - Brasil
    //      Núcleo de Neurociências

//===========================================================
========

    //float[] locmax;
    double[] locmax;
    int[] temp;
    int[] temp1;
    int[] maxidxs;
    int a;
    double [] bound;

    N=N-1;
    //locmax = new float [ff.length];
    locmax = new double [ff.length];
    for (int i=0; i<ff.length; i++) {
        locmax[i] = 0.0;
    }

    for (int i =1; i<locmax.length-1; i++) {
        if ((ff[i-1]<ff[i]) && (ff[i]>ff[i+1])) {
            //locmax[i] = (float) ff[i];
            locmax[i] = ff[i];
        }
    }

    N = min(N, locmax.length);
    temp1 = new int [N];

    temp    =    reverse(argsort(locmax));

    for (int i=0; i<N; i++) {
        temp1[i] = temp[i];
    }

    maxidxs = sort(temp1);
```

```
    //middle point between consecutive maxima
    bound = new double [N];
    for (int i =0; i <N; i++) {
        bound[i] = 0;
    }

    for (int i =0; i<N; i++) {
        if (i == 0) {
            a = 0;
        } else {
            a = maxidxs[i-1];
        }
        bound[i] = (a + maxidxs[i])/2;
    }
    return(bound);
}

double [][] EWT_Meyer_FilterBank(double [] boundaries, int Nsig) {
    //===========================================================
    // def EWT_Meyer_FilterBank(boundaries,Nsig):
    //===========================================================
    //      function mfb=EWT_Meyer_FilterBank(boundaries,Nsig)
    //
    //        This function generate the filter bank (scaling function + wavelets)
    //        corresponding to the provided set of frequency segments
    //
    //        Input parameters:
    //            -boundaries: vector containing the boundaries of frequency segments (0
    //                          and pi must NOT be in this vector)
    //            -Nsig: signal length
    //
    //        Output:
    //            -mfb: cell containing each filter (in the Fourier domain), the scaling
    //                  function comes first and then the successive wavelets
    //
    //        Author: Jerome Gilles
    //        Institution: UCLA - Department of Mathematics
    //        Year: 2012
    //        Version: 1.0
    //
    //        Python Version: Vinícius Rezende Carvalho - vrcarva@ufmg.br
    //        Universidade Federal de Minas Gerais - Brasil
    //        Núcleo de Neurociências
    //===========================================================

    int Npic;
    double gamma;
    double r;
    int Mi;
    double[] temp1;
    double[] temp2;
    double[] w;
    double [] aw;
    double an;
```

```
double pbn;
double mbn;
double[] yms1;
double [] yms2;
double[][] mfb;
double[] mfb_temp1;
double[] mfb_temp2;

Npic = boundaries.length;
//compute gamma
gamma = 1.0;
for (int k=0; k <Npic-1; k++) {
   r = (boundaries[k+1]-boundaries[k]) / (boundaries[k+1]+boundaries[k]);
   if (r < gamma) {
      gamma = r;
   }
}
r = (PI - boundaries[Npic-1]) / (PI + boundaries[Npic-1]);

if (r<gamma) {
   gamma = r;
}
gamma = (1.0-1.0/Nsig)*gamma;
//this ensure that gamma is chosen as strictly less than the min

mfb = new double [Nsig][Npic+1];
for (int i =0; i <Nsig; i++) {
   for (int j =0; j < Npic+1; j++) {
      mfb[i][j] = 0;
   }
}

//EWT_Meyer_Scaling
Mi=int(floor(Nsig/2));

//temp1 = new float[Nsig];
temp1 = new double[Nsig];
temp2 = new double[Nsig];

double div = (2*PI-2*PI/Nsig)/(Nsig-1);

for (int i=0; i<Nsig; i++) {
   temp1[i] = 0 + i * div;
}


for (int i=0; i<Nsig; i++) {
   temp2[i] = temp1[i];
}

w = fftshift_d(temp2);

double factor = -2.0 * PI;
```

```
    for (int i=0; i<Mi; i++) {
        w[i] = w[i] + factor;
    }

    aw = new double [Nsig];
    for (int i=0; i<Nsig; i++) {
        aw[i] = abs_double(w[i]);
    }

    yms1 = new double [Nsig];

    for (int i=0; i<Nsig; i++) {
        yms1[i] = 0;
    }

    an=1.0/(2*gamma*boundaries[0]);
    pbn=(1.0+gamma)*boundaries[0];
    mbn=(1.0-gamma)*boundaries[0];

    for (int k=0; k<Nsig; k++) {
        if (aw[k]<=mbn) {
            yms1[k]=1;
        } else if ((aw[k]>=mbn) && (aw[k]<=pbn)) {
            yms1[k]=(PI*EWT_beta(an*(aw[k]-mbn))/2);
            yms1[k] = cos((float) yms1[k]);
        }
    }

    yms2 = ifftshift_d(yms1);

    for (int i=0; i<Nsig; i++) {
        mfb[i][0] = abs((float)yms2[i]);
    }

    //generate rest of the wavelets
    for (int k=0; k<Npic-1; k++) {
        for (int i=0; i<mfb.length; i++) {
            mfb_temp1 = EWT_Meyer_Wavelet(boundaries[k], boundaries[k+1], gamma, Nsig);
            mfb[i][k+1] = mfb_temp1[i];
        }
    }

    for (int i=0; i <mfb.length; i++) {
        mfb_temp2 = EWT_Meyer_Wavelet(boundaries[Npic-1], PI, gamma, Nsig);
        mfb[i][Npic] = mfb_temp2[i];
    }
    return(mfb);
}

double EWT_beta(double x) {
    //
=================================================
    // def EWT_beta(x):
    // ================================================
```

```
    //      Beta = EWT_beta(x)
    //      function used in the construction of Meyer's wavelet
    // ===================================================

    double bm;

    if (x<0) {
        bm=0;
    } else if (x>1) {
        bm=1;
    } else {
        bm=(pow((float)x, 4))*(35.0-84.0*x+70.*(pow((float)x, 2))-20.*(pow((float)x, 3)));
    }
    return(bm);
}

double [] EWT_Meyer_Wavelet(double wn, double wm, double gamma, int Nsig) {
    //
===================================================
    // def EWT_Meyer_Wavelet(wn,wm,gamma,Nsig):
    //
===================================================
    //      ymw=EWT_Meyer_Wavelet(wn,wm,gamma,N)
    //
    //      Generate the 1D Meyer wavelet in the Fourier
    //      domain associated to scale segment [wn,wm]
    //      with transition ratio gamma
    //
    //      Input parameters:
    //          -wn : lower boundary
    //          -wm : upper boundary
    //          -gamma : transition ratio
    //          -N : number of point in the vector
    //
    //      Output:
    //          -ymw: Fourier transform of the wavelet on the band [wn,wm]
    //
    //      Author: Jerome Gilles
    //      Institution: UCLA - Department of Mathematics
    //      Year: 2012
    //      Version: 1.0
    //
    //      Python Version: Vinícius Rezende Carvalho - vrcarva@ufmg.br
    //      Universidade Federal de Minas Gerais - Brasil
    //      Núcleo de Neurociências

//===================================================
    int Mi;
    double [] temp;
    double [] aw;
    double [] ymw;
    //Complex [] ymw2_comp;
    double [] ymw2;
    //Complex [] ymw_comp;
```

```
double[] w;
double an;
double am;
double pbn;
double mbn;
double pbm;
double mbm;

Mi=int(floor(Nsig/2));

temp = new double [Nsig];
double div = (2*PI-2*PI/Nsig)/(Nsig-1);

for (int i=0; i<Nsig; i++) {
   temp[i] = 0 + i * div;
}

w = fftshift_d(temp);

double factor = -2.0 * PI;
for (int i=0; i<Mi; i++) {
   w[i] = w[i] + factor;
}

aw = new double [Nsig];
for (int i=0; i<Nsig; i++) {
   aw[i] =   w[i];
}
for (int i=0; i<Mi; i++) {
   aw[i] = abs((float) w[i]);
}

ymw = new double [Nsig];
for (int i=0; i<Nsig; i++) {
   ymw[i] = 0.0;
}

an=1.0/(2.0*gamma*wn);
am=1.0/(2.0*gamma*wm);
pbn=(1.0+gamma)*wn;
mbn=(1.0-gamma)*wn;
pbm=(1.0+gamma)*wm;
mbm=(1.0-gamma)*wm;

for (int k=0; k < Nsig; k++) {
   if ((aw[k]>=pbn) && (aw[k]<=mbm)) {
      ymw[k]=1;
   } else if ((aw[k]>=mbm) && (aw[k]<=pbm)) {
      ymw[k] =   cos((float) (PI*EWT_beta(am*(aw[k]-mbm))/2));
   } else if ((aw[k]>=mbn) && (aw[k]<=pbn)) {
      ymw[k] =   sin( (float) (PI*EWT_beta(an*(aw[k]-mbn))/2));
   }
}
```

```
  ymw2 = ifftshift_d(ymw);

  return(ymw2);
}

public static int[] argsort(final double[] a) {
  return argsort(a, true);
}

public static int[] argsort(double[] a, boolean ascending) {
  Integer[] indexes = new Integer[a.length];
  for (int i = 0; i < indexes.length; i++) {
    indexes[i] = i;
  }
  Arrays.sort(indexes, new Comparator<Integer>() {
    @Override
      public int compare(final Integer i1, final Integer i2) {
      return (ascending ? 1 : -1) * Double.compare(a[i1], a[i2]);
    }
  }
  );
  return asArray(indexes);
}

public static <T extends Number> int[] asArray(final T... a) {
  int[] b = new int[a.length];
  for (int i = 0; i < b.length; i++) {
    b[i] = a[i].intValue();
  }
  return b;
}

float[] array_square(float _array[]) {
  float _array2[];
  _array2 = new float [_array.length];

  for (int i=0; i<_array.length; i++) {
    _array2[i] = _array[i] * _array[i];
  }
  return _array2;
}

Complex[] fftshift(Complex _complex[]) {
  Complex complex2[];
  complex2 = new Complex [_complex.length];
  for (int i=0; i<_complex.length/2; i++) {
    complex2[_complex.length/2+i] = _complex[i];
  }
  for (int i=_complex.length/2; i<_complex.length; i++) {
    complex2[i-_complex.length/2] = _complex[i];
  }
  return complex2;
}
```

```
Complex[] ifftshift(Complex _complex[]) {
   Complex complex2[];
   complex2 = new Complex[_complex.length];
   for (int i=0; i<_complex.length/2; i++) {
      complex2[_complex.length/2+i] = _complex[i];
   }
   for (int i=_complex.length/2; i<_complex.length; i++) {
      complex2[i-_complex.length/2] = _complex[i];
   }
   return complex2;
}

double[] fftshift_d(double _double[]) {
   double double2[];
   double2 = new double[_double.length];
   for (int i=0; i<_double.length/2; i++) {
      double2[_double.length/2+i] = _double[i];
   }
   for (int i=_double.length/2; i<_double.length; i++) {
      double2[i-_double.length/2] = _double[i];
   }
   return double2;
}

double[] ifftshift_d(double _double[]) {
   double double2[];
   double2 = new double[_double.length];
   for (int i=0; i<_double.length/2; i++) {
      double2[_double.length/2+i] = _double[i];
   }
   for (int i=_double.length/2; i<_double.length; i++) {
      double2[i-_double.length/2] = _double[i];
   }
   return double2;
}

double[] append_double(double [] array1, double [] array2) {
   double [] array;
   array = new   double [array1.length+array2.length];
   for (int i = 0; i < array1.length; i++) {
      array[i] = array1[i];
   }
   for (int i = 0; i < array2.length; i++) {
      array[i+array1.length] = array2[i];
   }
   return(array);
}


Complex[] array_transpose(Complex _array[]) {
   Complex array2[];
   array2 = new Complex [_array.length];
   for (int j=0; j<_array.length; j++) {
      array2[j]=   _array[j];
```

```
    }
    return array2;
}

double[] array_transpose_double(double _array[]) {
    double array2[];
    array2 = new double[_array.length];
    for (int j=0; j<_array.length; j++) {
        array2[j]=    _array[j];
    }
    return array2;
}

Complex array_dot(Complex _array1[], Complex _array2[]) {
    Complex dot_result = new Complex(0, 0);
    for (int i=0; i<_array1.length; i++) {
        dot_result = dot_result.add(_array1[i].multiply(_array2[i]));
    }
    return dot_result;
}

double array_dot_double(double _array1[], double _array2[]) {
    double dot_result = 0;
    for (int i=0; i<_array1.length; i++) {
        dot_result += (_array1[i]*_array2[i]);
    }
    return dot_result;
}

Complex array_sum(Complex _array1[]) {
    Complex sum_result = new Complex(0, 0);
    for (int i=0; i<_array1.length; i++) {
        sum_result = sum_result.add(_array1[i]);
    }
    return sum_result;
}

double array_sum_double(double _array1[]) {
    double sum_result = 0;
    for (int i=0; i<_array1.length; i++) {
        sum_result += _array1[i];
    }
    return sum_result;
}

double abs_double(double _uDiff) {
    double _temp;
    if (_uDiff >= 0) {
        _temp = _uDiff;
    } else {
        _temp = - _uDiff;
    }
    return _temp;
}
```
