# Supplementary material for: Wavelet transform-based mode decomposition for EEG signals under general anesthesia
Source: PeerJ. 2024 Nov 15;12:e18518. doi: 10.7717/peerj.18518 (PMC11572389; doi:10.7717/peerj.18518)
Supplement: Supplemental Information 13 — The objective variable was the median value of the BIS from 10 patients and the explanatory variables were the statistically significant median values of the central frequencies and the TPs of the IMFs in A) VMD, B) the EWT, C) WMD, or D) VMD+EWT+WMD. The EEG data were obtained from the last 30 min before emergence in 10 patients who received sevoflurane GA. MAE: mean absolute error; RMSE, root mean squared error; ∗p < 0.05. [file peerj-12-18518-s013.pdf]

TABLE S1. MLR analysis of the BIS values and the parameters of the IMFs in VMD, the EWT, or WMD.

		**A.** VMD	**B.** EWT	**C.** WMD
explanatory variables: 6 central frequencies and 6 total powers				
determination factor		0.819	0.608	0.897
MAE		0.331	0.473	0.244
RMSE		0.426	0.626	0.321
y-intercept		9.707e-17	-6.513e-16	3.867e-16
explanatory variables			regression coefficient (*P*>|t|)	
central frequency	IMF-1	-	0.2458 (0.000*)	0.0803 (0.034*)
	IMF-2	-	-	-
	IMF-3	-	0.3469 (0.000*)	-0.0714 (0.014*)
	IMF-4	-	0.2336 (0.004*)	-
	IMF-5	-	-	0.1926 (0.000*)
	IMF-6	0.4614 (0.000*)	-	0.1535 (0.006*)
total power	IMF-1	0.1147 (0.005*)	-	-
	IMF-2	-	-0.2325 (0.000*)	-0.2519 (0.000*)
	IMF-3	-	--	-
	IMF-4	-0.4115 (0.000*)	0.1493 (0.009*)	-0.2260 (0.000*)
	IMF-5	-	0.2447 (0.000*)	-
	IMF-6			0.3144 (0.000*)
		**D.** VMD+EWT+ WMD		
determination factor			0.879	
MAE			0.260	
RMSE			0.348	
y-intercept			-2.44e-16	
			regression coefficient (*P*>|t|)	
central frequency	IMF-1	-	0.1021(0.011*)	-
	IMF-2	0.0897 (0.004*)	-	-
	IMF-3	-	-	-0.0838 (0.010*)
	IMF-4	-	0.1642(0.000*)	-
	IMF-5	-	-	-
	IMF-6	-	-	0.1958 (0.000*)
total power	IMF-1	-	-	-
	IMF-2	-	-	-0.2762 (0.000*)
	IMF-3	-	-	-
	IMF-4	-	0.1197 (0.001*)	-0.3080(0.000*)
	IMF-5	-	0.1000 (0.001*)	-
	IMF-6	-	-	-

The objective variable was the median value of the BIS from 10 patients and the explanatory variables were the statistically significant median values of the central frequencies and the TPs of the IMFs in **A)** VMD, **B)** the EWT, **C)** WMD, or **D)** VMD+EWT+WMD. The EEG data were obtained from the last 30 mins before emergence in 10 patients who received sevoflurane GA. MAE: mean absolute error; RMSE: root mean squared error; *$p<0.05$.
