# Supplementary material for: Wavelet transform-based mode decomposition for EEG signals under general anesthesia
Source: PeerJ. 2024 Nov 15;12:e18518. doi: 10.7717/peerj.18518 (PMC11572389; doi:10.7717/peerj.18518)
Supplement: Supplemental Information 14 — The objective variable was the median value of the BIS from 10 patients, and the explanatory variables were (1) the six median values of the central frequencies and (2) the six total powers of the IMFs. The EEG data were obtained from the last 30 min period before emergence in 10 patients who received sevoflurane GA. MAE, mean absolute error; RMSE, root mean squared error; ∗p < 0.05. [file peerj-12-18518-s014.pdf]

TABLE S2. MLR analysis using the BIS values and the parameters of the IMFs in VMD, the EWT, or WMD.

		VMD	EWT	WMD
**1)** explanatory variables: 6 median values of central frequencies				
determination factor		0.795	0.504	0.852
MAE		0.345	0.536	0.311
RMSE		0.452	0.705	0.385
y-intercept		9.707e-17	-6.513e-16	3.867e-16
			regression coefficient (*P*>|t|)	
central frequency	IMF-1	-0.1413 (0.000*)	-0.3218 (0.000*)	-0.0225 (0.583)
	IMF-2	0.0649 (0.137)	-0.1157 (0.100)	0.0170 (0.523)
	IMF-3	0.1274 (0.031*)	0.4080 (0.000*)	-0.1011 (0.003*)
	IMF-4	-0.0763 (0.418)	0.1171 (0.164)	0.1063 (0.009*)
	IMF-5	0.1842 (0.058)	0.0074 (0.901)	0.3705 (0.000*)
	IMF-6	0.6057 (0.000*)	0.0980 (0.087)	0.4213 (0.000*)
**2)** explanatory variables: 6 median values of total powers				
determination factor		0.799	0.369	0.876
MAE		0.358	0.593	0.27
RMSE		0.448	0.795	0.352
y-intercept		9.707e-17	-6.513e-16	3.867e-16
			regression coefficient (*P*>|t|)	
total power	IMF-1	0.2795 (0.000*)	0.1048 (0.091)	0.0270 (0.336)
	IMF-2	0.0071 (0.864)	-0.4243 (0.000*)	-0.3204 (0.000*)
	IMF-3	-0.2190 (0.000*)	-0.3045 (0.000*)	-0.0565 (0.383)
	IMF-4	-0.4198 (0.000*)	0.0164 (0.814)	-0.3409 (0.000*)
	IMF-5	-0.0381 (0.414)	0.1030 (0.180)	0.0311 (0.490)
	IMF-6	-0.1292 (0.002*)	0.0716 (0.334)	0.4213 (0.000*)

The objective variable was the median value of the BIS from 10 patients, and the explanatory variables were **1)** the six median values of the central frequencies and **2)** the six total powers of the IMFs. The EEG data were obtained from the last 30 min period before emergence in 10 patients who received sevoflurane GA. MAE: mean absolute error; RMSE: root mean squared error; *$p<0.05$.
